# Transcranial Electrical Stimulation Offers the Possibility of Improving Teamwork Among Military Pilots: A Review

**DOI:** 10.3389/fnins.2022.931265

**Published:** 2022-07-13

**Authors:** Hongliang Lu, Yajuan Zhang, Peng Huang, Yan Zhang, Sizhe Cheng, Xia Zhu

**Affiliations:** Faculty of Medical Psychology, Air Force Medical University, Xi’an, China

**Keywords:** teamwork, hyper-scanning, tACS, IBS, fNIRS, military pilot

## Abstract

Effective teamwork among military pilots is key to successful mission completion. The underlying neural mechanism of teamwork is thought to be inter-brain synchronization (IBS). IBS could also be explained as an incidental phenomenon of cooperative behavior, but the causality between IBS and cooperative behavior could be clarified by directly producing IBS through extra external stimuli applied to functional brain regions. As a non-invasive technology for altering brain function, transcranial electrical stimulation might have the potential to explore whether top-down enhancement of the synchronization of multiple brains can change cooperative behavioral performance among members of a team. This review focuses on the characteristic features of teamwork among military pilots and variations in neuroimaging obtained by hyper-scanning. Furthermore, we discuss the possibility that transcranial electrical stimulation could be used to improve teamwork among military pilots, try to provide a feasible design for doing so, and emphasize crucial aspects to be addressed by future research.

## Introduction

With the rapid development of network information technology and the deepening of data resource sharing, the mode of modern war has become one of combined arms strategies implemented through multi-unit cooperation. The use of electronic countermeasures and stealth operations make combat missions more complex and changeable, making it difficult for a single combat unit (such as a single soldier or a single aircraft) to be competent for all relevant tasks. Instead, effective operation must involve the cooperation of combat units (Min, 2013). The premise of coordination is to form a combat team with team members as the core. So long as they operate as a “team” rather than as a “group,” synergistic benefits can be achieved at lower cost, such that “1 + 1 > 2.” Generally, modern air force combat units always take the form of action teams consisting of two or more aircraft (Ohlander et al., 2016a). The level of coordination between team members in air battle has been found to play an important role in the successful completion of a military mission, and it is significantly positively correlated with team performance (Ohlander et al., 2009). In recent years, there has arisen not only human–human team cooperation, but also human–machine cooperation, which has rapidly developed into a new mode of combat (Stowers et al., 2021). Such a hybrid team similarly provides a combination of human decision-making and a machine information sharing chain, greatly improving the effectiveness of air combat (Jian, 2017). Teamwork thus plays an important role in military flight operations, and it is of great significance to maximize team cooperation in order to achieve military objectives. It is particularly important to find a way to effectively improve teamwork in both peacetime and wartime. As a representative non-invasive brain intervention technology, transcranial electrical stimulation has been proven to improve individual cognitive functions such as attention, execution, and risk decision-making by changing neuronal excitability or inducing neural synchronization and oscillation through low-intensity current (Guo et al., 2018; Kronberg et al., 2020; Lipka et al., 2021; Lu et al., 2021, 2020). Davis and Smith (2019) have discussed the risks and benefits of transcranial electrical stimulation technology in military applications and has affirmed the military advantages of transcranial electrical stimulation (such as cognitive improvement in combat, enhancement of survivability for emergency, and so on), believing that this technology could have a great potential in improving military combat effectiveness in the future. Improving the cognitive ability of individual soldiers might have a positive impact on teamwork. However, studies of teams often require a holistic analysis of individuals in a collaborative context, and exploring possible roles for transcranial electrical stimulation in intra-team cooperation is critical for both current needs and military preparedness. It has been proven that transcranial electrical stimulation technology cannot only enhance cognitive ability but also have different degrees of positive impact on social interactions between multiple individuals (Peled-Avron et al., 2019; Pan et al., 2021). Although the range of studies has been limited, the conclusions of the existing studies indicate that transcranial electrical stimulation technology could be a crucial way to improve the capabilities of military pilot teams in the future.

This review summarizes and discusses prior relevant studies, which are divided into the following groups. First, we introduce the concept and features of military pilot teamwork. Second, imaging studies on the potential neural mechanisms of teamwork among military pilots are summarized. Third, this paper reviews the research on the improvement of teamwork among military pilots, and in particular the effect of transcranial electrical stimulation technology on improving teamwork. Finally, we indicate the limitations of current research and propose future prospects for the improvement of teamwork in military pilots by transcranial electrical stimulation.

## Concept and Features of Military Pilot Teamwork

**FIGURE 1:**
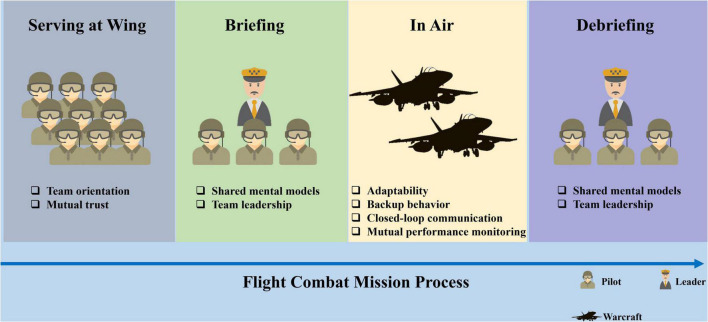
Teamwork factors required at different flight combat mission stages. Adapted from Ohlander et al. (2019).

As the basic unit of an organization, a team is composed of two or more individuals. In order to achieve a common team goal, team members maximize the team’s benefits through orderly division of labor. This form of organization plays an important role in the survival of animals in nature and in the operation of human social activities (Salas et al., 1992; Anderson, 2001). Not only lions and wolves (Scheel and Packer, 1991; Anderson, 2001; Pennisi, 2017), but also medical and military teams are all typical teams (Doyle et al., 2020; Martin et al., 2022). Salas et al. (2005) put forward the “Big Five” model of teamwork on the basis of prior studies. This model contains five core factors (team leadership, mutual performance monitoring, backup behavior, adaptability, and team orientation) and three additional factors (shared mental model, closed-loop communication, and mutual trust). This model has enjoyed wide support over the past decade, and it has been studied and applied in various fields such as medical treatment, rescue, aviation, the military, and air traffic control (Driskell et al., 2018; Svensson et al., 2020). Thus far, it has been verified that the teamwork model proposed by Salas et al. (2005). is applicable to teams of military pilots (Ohlander et al., 2018). Ohlander et al. (2019) integrated the five core elements of the “Big Five” teamwork model with the three coordinating factors and found that the importance of mutual performance monitoring, closed-loop communication, shared mental model, adaptability, mutual trust, team orientation, team leadership, and backup behavior decreased in turn, after interviewing a group of experienced active fighter pilots. Further research found that fighter pilot teamwork should be analyzed within a full mission cycle, which includes building the flight team before a mission (choosing members, appointing a team leader, task allocation, etc.), team discussion (division of labor, implementation rules, etc.), performance of the flight mission, reflection and discussion after the mission, and finally team dissolution (Ohlander et al., 2016a,b). As shown in Figure 1, the importance of the factors at each stage is different. At the beginning of team building, mutual trust and team orientation are most important, as they are the prerequisite for the successful completion of team tasks. In the early stages of the task, group members often discuss and exchange ideas, familiarize themselves with the task process, and establish a shared mental model. The stage of task execution requires close communication between team members, backup behavior, mutual performance monitoring, and adaptability for emergencies. After the task, a meeting is held to update, based on experience, the existing shared mental model.

In summary, military pilots often fly as a team to successfully complete military missions. The “Big Five” team model can explain the weights of different factors at different stages of a mission, but the interviews were based only on subjective data. They lack the support of biological evidence, which limits the possibilities for in-depth understanding of the occurrence and development of team cooperation. The essence of collaboration among team members is still a complex social interaction behavior, including the most basic cooperation, interpersonal learning, trust, etc. Such social interaction between individuals has been proven to be key to the success of teamwork (Lechler, 2001). Therefore, using mature neuroimaging technology to explore the neural mechanisms of team cooperation can provide a valuable reference to reasonably adjust the team structure, cooperation strategy, and team member training.

## Neuroimaging Studies on the Potential Neural Mechanisms of Teamwork in Military Pilots

Team behavior among military pilots is a type of social interaction: in essence, information exchange and sharing between individual nervous systems. However, some information is lost due to natural physical isolation during transmission, such as sense perceptions, behavior, language, etc. (Kingsbury and Hong, 2020). Therefore, the study of teamwork among military pilots should not be limited to the observation of behavioral performance. In order to obtain more adequate information, we need to explore the neural mechanisms underlying teamwork. Previous imaging studies in cognitive neuroscience have usually focused on a single individual or single brain – for example, a cognitive function or emotional response that is accompanied by changes in the activation of a specific brain region or changes in the functional connectivity of multiple brain regions. Neuroimaging studies of multiple individuals in groups have only emerged in the last two decades. Hyper-scanning refers to a technology that can support real-time signal transmission, recording, and analysis between two or more brains, which can be used to explore the neural mechanisms of social interaction (Hari and Kujala, 2009; Nguyen et al., 2021a). Functional magnetic resonance imaging (fMRI), electroencephalography (EEG), functional near-infrared spectroscopy (fNIRS), and other brain imaging techniques can be used for hyper-scanning studies (Czeszumski et al., 2020). Blood oxygenation level dependent (BOLD) signals of cerebral blood flow are used in fMRI to perform tomography with high spatial accuracy but low temporal resolution; this technology indirectly reflects the activity of brain neurons. Hyper-scanning studies by fMRI to date have lacked real social interaction and sufficient ecological validity. Such studies are generally designed around subjects’ subjective imagination (Shibata et al., 2011), network communication between subjects, etc. (Redcay et al., 2010). Compared with fMRI, EEG has had a wider application in hyper-scanning through the real-time acquisition of EEG signals from multiple individuals with high temporal resolution, which can capture transient electrophysiological signals of brain activities under rapid stimulation. However, the weak spatial resolution of traditional EEG cannot accurately observe the activation of brain regions, and limited movement tolerance weakens the possibility of using such an experimental design in real activities (Liu et al., 2018). Fortunately, the recent studies have already shown that electrophysiological source imaging (ESI) based on EEG would provide improved spatiotemporal precision for further application of EEG (He et al., 2018; Edelman et al., 2019; Seeber et al., 2019), and that motion artifacts of EEG could be promisingly rejected by using dry flexible electrodes, in-ear EEG, optimized algorithms of signal processing and so on (Seok et al., 2021). fNIRS has been applied for recording hemoglobin concentrations in particular brain regions by near-infrared light; it has been widely used in the study of infant neurodevelopment due to its strong tolerance for movement (Teresa and Marisa, 2015). Thus, fNIRS will play an important role in the future of social interaction studies thanks to its moderate spatial resolution, temporal resolution, motion tolerance, and portable operation (Mayseless et al., 2019; Reindl et al., 2019; Li et al., 2021). Therefore, a teamwork neuroimaging study based on fNIRS hyper-scanning will be emphasized here. It’s worth mentioning that optically pumped magnetometers (OPMs) enabled wearable magnetoencephalography (MEG) is a new and competitive approach to assess brain function (Boto et al., 2018), which would be considered have potential to provide a guidance for the neural mechanisms of teamwork in the future.

Inter-brain synchronization (IBS) usually occurs when individuals in a social interaction have shared behaviors or intentions (i.e., cooperation) (Mayseless et al., 2019). Generally, the index for assessing IBS is coherence calculated by oxy-hemoglobin (HbO) concentration which has higher signal-to-noise ratio than deoxy-hemoglobin (HbR) concentration in fNIRS studies, and so IBS is also referred to as interpersonal brain coherence (Cui et al., 2012). IBS is an indicator of the degree of consistency of brain activity, obtained by hyper-scanning two or more individuals in a group (Xu et al., 2012). IBS of the bilateral dorsolateral prefrontal cortex during cooperative behavior among team members is stronger than that during competitive behavior, and such a synchronization effect increases over time (Lu and Hao, 2019). Liu et al. (2021) conducted a nine-person drumming experiment with three experimental modes: random drumming, group focus drumming, and metronome focus drumming. They found that the self-reported interdependence was higher in the group focus drumming mode and was accompanied by higher IBS of the temporoparietal junction and the medial prefrontal cortex, representing an understanding of others’ thoughts and intentions. These results provided imaging evidence for the important role of shared mental models in team cooperation. Interestingly, team creativity was higher in the cooperative condition than in the competitive condition, and the increased creativity was associated with enhanced IBS of the right dorsolateral prefrontal cortex and the right temporo-parietal lobe (Lu et al., 2019). Therefore, IBS of functional brain regions seems to be a potential neural mechanism of teamwork and is closely associated with team creativity. In addition, IBS may be influenced by factors such as intimacy, gender, profession, social experience, etc. IBS between father and child in the bilateral dorsolateral prefrontal cortex and the left temporoparietal junction were significantly increased during cooperative tasks (Nguyen et al., 2021b). A prior study of IBS between mother and child further showed that children’s responsiveness can promote their commitment compliance through the mediating effect of IBS of the temporoparietal junction (Zhao et al., 2021). This evidence indicates that the enhancement of IBS in the corresponding brain region is promoted by a healthy parent-child relationship, which is of great significance for the psychological development of children. IBS could be affected by gender, in that the IBS of the prefrontal lobe is higher in heterosexual cooperation than in homosexual cooperation, and this neural synchronization is directional (female to male) (Cheng et al., 2015; Pan et al., 2017). Occupation is also one of the factors that influence IBS. Athletes majoring in team sports have shown better cooperative behaviors than other subjects, accompanied by significant IBS in the dorsolateral prefrontal region (Li et al., 2020). Individuals whose social experiences differed from each other had better cooperative behavior and greater IBS than those with similar social experiences (Sun et al., 2021). Meanwhile, the team creativity of individuals with low creativity was equal to that of individuals with high creativity, and IBS intensity of the frontal lobe of the former was higher than that of the latter (Hua et al., 2018).

An enhancement of IBS during cooperative behavior between pilots was also observed in previous studies. Similar to Ohlander et al.’s (2016b) evaluation of changes in the core elements of fighter pilot teamwork during flight, IBS in functional brain regions also changed at different stages of flight tasks. It has been found, when using scanning technology for real-time monitoring of brain signals of each of two pilots during a simulated flight mission, that IBS of the frontal and parietal cortex calculated by EEG signals in alpha or theta band, was strongest when two pilots fly in the most difficult phases (take-off and landing) requiring the highest level of cooperation, and that IBS of the frontal and parietal cortex was weak or even zero in the other process of flight (Astolfi et al., 2012; Toppi et al., 2016). Therefore, IBS in functional brain regions seems to be a valid neural indicator of teamwork. However, it should be noted that these indicators have merely been shown to accompany cooperative tasks, and whether they could be used as a scientific explanation of cooperative behavior remains to be further determined. Classic cognitive neuroscience studies have a similar limitation in that correlations between time-dependent behavioral changes and neurological indicators cannot be used as a basis for causal inference. This question will be discussed in detail later.

## Research on the Improvement of Teamwork Among Military Pilots

Effective teamwork has been proven to play an important role to deal with unexpected instances in the public health sector’s response to the COVID-19 crisis (Tomer et al., 2021). The same is true for military pilots, and the question of how to ensure strong teamwork to maximize the effectiveness of the team is particularly crucial. At present, research on the improvement of teamwork can broadly be classified into optimization of team structure, improvement of communication among members, skill training, motivation, and enhancement of brain area function.

Team structure is extremely important for the whole team, and a reasonable team structure can often determine whether a task is successfully completed. For example, a medical team in the intensive care unit mainly includes attending doctors, medical interns, nurses, pharmacists, dietitians, and other staff members. Only with the complementary advantages of these staff can teamwork be maximized and the safety of critically ill patients be guaranteed Coleman and Pon (2013). In addition, the team as a whole should be established on the premise of effective communication around shared team goals or mental models, as effective communication between members can ensure that information is fully and accurately transmitted within the team. One study has found that the communication ability of team members was significantly improved, resulting in increased satisfaction of their patients, after communication training in an outpatient environment (Dodge et al., 2019). With regard to skill training, a team member not only contributes to the common goal, but also gives full play to one’s unique advantage. Therefore, professional skill training not only improves individual ability unilaterally, but also reduces the probability of weaknesses of the team. Motivation factors have also been found to play a key role in the application of team training to improve teamwork (Tabassi et al., 2012). All of the above are classic behavioral methods, which directly promote teamwork behavior by changing the external performance of individuals or the whole team. Cognitive neuroscience generally believes that stable changes in behavior depend on variations in neural mechanisms, but the aforementioned methods promote teamwork by changing the environment (i.e., team structure) or behavioral habits (i.e., communication, skills, etc.), rather than directly intervening in the target brain regions. In addition, this kind of method requires more training resources, training time, experience, etc. Based on the aforementioned studies of neuroimaging related to teamwork behavior, IBS appears to be the underlying neural mechanism of teamwork. Therefore, we suspect that the synchronization of neural oscillations between members supports the occurrence and development of collaborative behaviors. Can IBS in the corresponding brain regions of individuals be changed by external intervention, and can teamwork be affected thereby? In theory, such a top-down approach is easy to implement by using non-invasive brain stimulation technology. This can help us to solve two problems: proving the causal relationship between IBS and cooperation behavior, quantifying the neural and behavioral benefits induced by external stimuli, and exploring the promoting effects of different stimulus parameters on teamwork.

### Concept, Classification, and Characteristics of Transcranial Electrical Stimulation

Transcranial electrical stimulation is a safe, non-invasive technology that delivers low-intensity current to the cerebral cortex to change brain functions by forming a current pathway through scalp electrodes (Reed and Cohen Kadosh, 2018). The clinical applications of transcranial electrical stimulation are extremely wide, extending to conditions such as compulsive behavior, migraine attack, dementia, Alzheimer’s, etc. (Brunoni et al., 2012; Khedr et al., 2019; Antal et al., 2020; Grover et al., 2021; Moussavi et al., 2021). Cognitive improvements in healthy individuals have also been observed after transcranial electrical stimulation (Metuki et al., 2012; Katz et al., 2017; Berger et al., 2018; Reinhart and Nguyen, 2019; Borwick et al., 2020). Transcranial electrical stimulation is divided into transcranial direct current stimulation (tDCS), transcranial alternating current stimulation (tACS), and transcranial random noise stimulation (tRNS). tDCS stimulates the target brain region with low-intensity direct current (0.5–2 mA) to change the excitability of neurons (Nitsche et al., 2002). tACS mainly induces synchronous oscillations of neurons in target brain regions through different frequency currents (Herrmann et al., 2016). tRNS, in a sense, is also a special “alternating current stimulation” to change neuronal excitability by delivering stimulation with random frequencies and amplitudes within a specific stimulation range, which has been proved to produce more promising benefit on auditory perception than other transcranial electrical stimulation (Fertonani and Miniussi, 2017; Prete et al., 2017, 2018). However, tDCS and tACS are most widely used in the study of cognitive improvement at present, so the subsequent introduction will mainly focus on these two methods. High-precision transcranial electrical stimulation has a current path composed of multiple electrodes and high directivity, so it has great advantages in stimulation accuracy and current density compared to traditional transcranial electrical stimulation (DaSilva et al., 2015; Turski et al., 2017; Pa Rlikar et al., 2021). In addition, the following parameters can affect the intervention effect in transcranial electrical stimulation experiments. First, the range of current intensities in transcranial electrical stimulation experiments is generally not higher than 2 mA; generally speaking, current intensities at the upper end of that range bring better intervention benefits. Second, the excitability of the cortex is inhibited under the cathode while increased under the anode during tDCS (Shin et al., 2015). Third, the selected brain region should be covered by the current field, which can be simulated using computer software (Lu et al., 2021, 2020). The last parameter is the frequency of tACS. In general, synchronous neural oscillations are more likely to occur when the stimulus frequency is consistent with the internal frequency of neurons in the functional brain regions (Herrmann et al., 2013; Takeuchi and Izumi, 2021).

### The Improvement of Teamwork by Transcranial Electrical Stimulation

**FIGURE 2:**
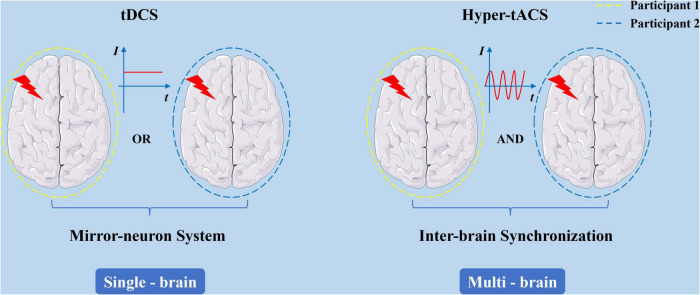
Different intervention patterns of tDCS and hyper-tACS in promoting cooperative behavior.

It has been found, according to the underlying neural mechanism of teamwork, that it is feasible to modulate brain function through external stimulation to change cooperative behavioral performance between team members. However, as shown in Figure 2, there are different intervention models between tDCS and tACS (hyper-tACS) for improving cooperative behavior. The main characteristic of IBS is synchronous nerve oscillations between multiple brains, while the stimulation of tDCS is characterized by direct current interference in a particular brain region, and seems unable to directly induce the synchronization of the corresponding brain region through neural entraining. Enticott et al. (2012) found that tDCS intervention of brain regions (inferior frontal gyrus) involved in the mirror neuron system in healthy individuals enhanced interpersonal motor resonance. The mirror neuron system plays an important role in imitation activities, interpersonal learning, and other behaviors (Oberman et al., 2007; Mainieri et al., 2013; Meng Yuan et al., 2018), and autism is considered in part to be related to dysfunction in the mirror neuron system (Hamilton, 2008). A previous study has shown that cathode tDCS significantly reduced musicians’ assessment of musical creativity, which was related to the mediating effect of empathy (Colombo et al., 2021). Anodal tDCS intervention on the right inferior frontal gyrus of healthy subjects is thought to induce imitative behavior in social interaction (Hogeveen et al., 2015). Therefore, although tDCS cannot directly regulate the neural oscillation rhythm in a particular brain region across multiple brains, it could modulate the cooperative behavior of subjects by intervening in the mirror neuron system, which is closely related to cooperation and teamwork. Additionally, as a crucial part in rapid instructed task learning related with teamwork (Meiran et al., 2016), working memory was also proved to be effectively improved by tDCS and other transcranial electrical stimulation, which would provide a way to enhance teamwork (Ke et al., 2019; Nissim et al., 2019; Zeng et al., 2022). However, the nature of cooperative behavior still probably lies in the occurrence and development of multi-brain IBS. Using tACS technology would provide more possibilities for future research into the influence of different frequency and phase parameters on teamwork behavior.

Novembre and Iannetti (2021a,b) argued that the phenomenon of multi-brain IBS observed by hyper-scanning cannot be clearly explained in relation to social interaction: is it actual causality, or mere contingency? Therefore, regulating interbrain synchronization directly through multi-brain stimulation (MBS), such as hyper-tACS, and taking IBS as an independent variable in the study is critical to understanding the neural mechanism of social interaction behaviors. Hyper-tACS is used to stimulate one or several functional brain regions to induce the coupling of neural oscillations between multiple brains, with the goal of promoting social interaction behaviors such as collaborative writing and interpersonal learning (Novembre et al., 2017; Pan et al., 2021). The effects modulated by hyper-tACS are always phase-frequency specific. A study found that 6 Hz in-phase hyper-tACS located on the prefrontal lobe could be successfully applied to induce spontaneous synchronous movement between teachers and students. A song teaching effect was also promoted, while interventions at other frequencies or phases did not produce similar effects (Pan et al., 2021). 20 Hz in-phase hyper-tACS on left motor cortex has been found to increase the synchronization of interpersonal movement, while the same results were not present for other frequencies or for anti-phase or false stimulation (Novembre et al., 2017). However, it has been found that such immature hyper-tACS technology does not produce significant changes in promoting synchronicity under the two-person drumming task, although this may be related to the choice of stimulus program (Szymanski et al., 2017). Therefore, hyper-tACS does provide a possibility for the improvement of teamwork or cooperation behavior, but there are still urgent problems to be solved in the future, such as the specific settings of parameters, selection of stimulus programs, synchronous imaging acquisition, compatibility of hardware and software, etc.

## Research Prospects for Transcranial Electrical Stimulation Technology to Promote Teamwork Among Military Pilots

**FIGURE 3:**
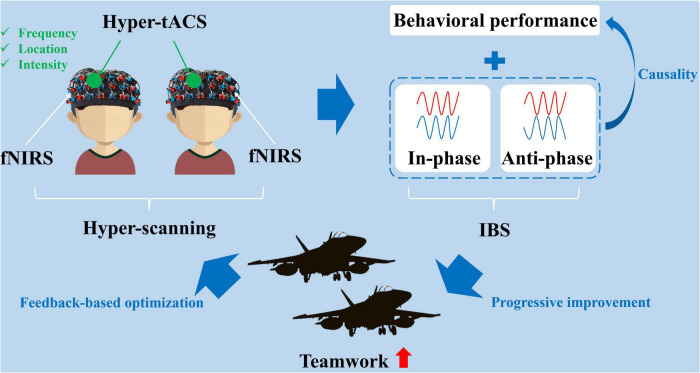
Possible technical routes for research on military pilot teamwork enhancement.

Based on the evaluation of team cooperation among military pilots and related enhancement technology, and taking into account the advantages and disadvantages of methods used in prior studies, this review puts forward possible design ideas for future research on teamwork among military pilots, as shown in Figure 3. As a neuroimaging technique suitable for multiple participants, hyper-scanning based on fNIRS should be used to record information about synchronous oscillation in the target brain regions. Next, the connection between behavioral performance during a task (under three conditions: cooperative, competitive, or neutral) and IBS should be analyzed before and after hyper-tACS intervention. The underlying causality would thus be clarified. In addition, the enhancement of military pilot teamwork could be explored based on a credible improvement strategy according to the effective parameters of hyper-tACS, as obtained by laboratory investigation. However, the details of the study design must be optimized by feedback, based on the benefits obtained.

Future studies should mainly focus on two aspects:

### Quantification of Behavior and Neural Mechanism in the Teamwork of Military Pilots

Although the teamwork environment of military pilots has characteristics such as high pressure, high noise, narrow scope of activities, high mental load, fast decision-making, and difficult situational awareness, teamwork among military pilots still conforms to the “Big Five” teamwork model. Therefore, despite being a special form of teamwork, military pilot teamwork is still a kind of social interaction. However, prior studies on social interaction behavior were still based on simple laboratory research. Military pilots are faced with a complex and changeable environment when performing tasks, and replicating that environment could be key to quantifying such cooperative behavior with improved accuracy and ecological validity. Virtual reality, simulated aircraft, and flight operation games are all new behavioral quantitative tools with high ecological validity (Bauer and Klingauf, 2006; Hans et al., 2016; Villafaina et al., 2021). Compared to the traditional laboratory paradigm, such tools could provide more vivid operating conditions and increased participation for participants. In addition, the selection of suitable neuroimaging tools (such as fNIRS) with great movement tolerance could ensure the collection and analysis of neuroimaging data in such an environment. Portable fNIRS would be more suitable since it is lighter, cheaper, wireless, and has better adaptability in most social scenes compared to traditional wire-based fNIRS (Agro et al., 2016; Gozde, 2017). In particular, fNIRS equipped on each member of the team would provide more comprehensive monitoring of brain regions in the study of interactions between pilots in military fighter formations. The data analysis method for fNIRS also must be selected carefully according to the actual conditions of study. In general, the common method is based on averaging the target signal during the time window before conducting wavelet coherence or Granger causality analysis (Hu et al., 2021), but time information would be lost because the tasks always undergo dynamic changes. This is problematic because different stages of a task are accompanied by different states of IBS. Therefore, a dynamic IBS analysis method would retain time-level information and explore dynamic changes in IBS over time (Li et al., 2021). Accurately quantifying behavioral performance and neuroimaging changes in military pilots during teamwork tasks would help us establish effective evaluation schemes and data sets, and improve the screening validity during team member selection. The difficulty to be overcome in future research would be to select appropriate evaluation methods and parameters to create the prerequisite conditions for follow-up interventions to promote teamwork.

### Selection of Hyper-Transcranial Alternating Current Stimulation Scheme to Promote Military Pilot Teamwork

Team structure, professional skills, motivation, and other factors are the most routine and basic approaches for the promotion of teamwork among military pilots (Zhiqiang, 2015; Aitoro, 2019). These methods are carried out from the early stages of pilot training. However, “tacit understanding” training among the members of the flying formation is absent, and this could cause a failure of cooperative behavior among pilots and increase the difficulty of task completion. Due to the tension of a training mission, military pilots cannot afford to spend time on interactive cooperative behavior training, but hyper-tACS could enable a military pilot to obtain high compatibility and more quickly adapt to their partner. The question of how to maximize the intervention effect is also worth exploring in future studies, especially with regard to the selection of stimulus sites, frequency, intensity, and phase of hyper-tACS. Some complex cognitive processes have been shown to be the result of cross-frequency coupling between brain regions (such as the inhibitory prefrontal cortex’s regulation of the motor cortex), so it may be necessary to adopt different frequency-coupled stimulus modes during hyper-tACS intervention (Riddle et al., 2021). In addition, there are lingering concerns about the safety of transcranial electrical stimulation. A large number of studies have shown that even repeated stimulation is safe and reliable compared with sham stimulation as long as the operational requirements of electrical stimulation were conducted in strict accordance with safety protocols (Turski et al., 2017; Nikolin et al., 2019). Choe et al. (2016) conducted electrical stimulation in the laboratory on 32 healthy subjects undergoing flight training to explore its influence on flight performance and the relevant data of EEG and fNIRS. Thus, the safety of transcranial electrical stimulation is guaranteed under proper operation. In view of the current model of air combat, how military pilots engage in optimal teamwork plays a key role in successful completion of the mission. Therefore, future research should focus on solving the problem of how to improve the teamwork behavior of military pilots using a plan that has been optimized based on feedback from the evaluation results.

## Conclusion

In this review, we have clarified the model of teamwork among military pilots and provided an underlying explanation for the neural mechanism of teamwork. However, although IBS is known to be closely related to cooperative behavior, the question of causality is not clear. Thus, we hypothesize that transcranial electrical stimulation could be applied to directly stimulate brain regions related to teamwork to enhance IBS among multiple members in a team, and the causal link between IBS and cooperative behavior would then be clarified. Furthermore, it is crucial for military pilots to improve their teamwork by either tDCS or hyper-tACS. We therefore provided a feasible study design as a basis for an enhancement strategy. The hyper-scanning and hyper-tACS could provide a possible way for military pilots to enhance their capability for teamwork and would help us better explore the relationship between synchronous oscillation and cooperative behavior. We hope this review can provide some theoretical inspiration for future research on improving the combat effectiveness of military pilot teams, and we put forward suggestions on the basis of current research to improve relevant study designs in the future.

## Author Contributions

HL and YjZ completed the writing of the manuscript and manuscript revision. PH, YnZ, and SC conducted the search and collation of literature. XZ provided the financial support and writing guidance. All authors contributed to the article and approved the submitted version.

## Conflict of Interest

The authors declare that the research was conducted in the absence of any commercial or financial relationships that could be construed as a potential conflict of interest.

## Publisher’s Note

All claims expressed in this article are solely those of the authors and do not necessarily represent those of their affiliated organizations, or those of the publisher, the editors and the reviewers. Any product that may be evaluated in this article, or claim that may be made by its manufacturer, is not guaranteed or endorsed by the publisher.

## References

[B1] Agro D. Canicattì R. Pinto M. Morsellino G. Tomasino A. Adamo G. (2016). “Design and implementation of a portable fnirs embedded system,” in *Applications in Electronics Pervading Industry, Environment and Society. Lecture Notes in Electrical Engineering*, Vol. 351 ed. De Gloria A. (Cham: Springer International Publishing), 43–45. 10.1007/978-3-319-20227-3_6

[B2] Aitoro J. (2019). *Army to Kick off Virtual Reality Pilot Trainning. Defense News.* https://www.militarytimes.com/land/2019/03/05/army-to-kick-off-virtual-reality-pilot-training-program-in-april/?contentQuery=%7B%22section%22%3A%22%2Fhome%22%2C%22exclude%22%3A%22%2Fnews%2Fyour-army%22%2C%22from%22%3A5%2C%22size%22%3A10%7D&contentFeatureId=f0fmoahPVC2AbfL-2-1-8doi: (accessed February, 2022).

[B3] Anderson C. (2001). Teams in animal societies. *Behav. Ecol.* 12 534–540. 10.1093/beheco/12.5.534

[B4] Antal A. Bischoff R. Stephani C. Czesnik D. Klinker F. Timäus C. (2020). Low intensity, transcranial, alternating current stimulation reduces migraine attack burden in a home application set-up: a double-blinded, randomized feasibility study. *Brain Sci*. 10:888. 10.3390/brainsci10110888 33233400 PMC7700448

[B5] Astolfi L. Toppi J. Borghini G. Vecchiato G. Babiloni F. (2012). “Cortical activity and functional hyperconnectivity by simultaneous EEG recordings from interacting couples of professional pilots,” in *Proceedings of the 2012 Annual International Conference of the IEEE Engineering in Medicine and Biology Society*, San Diego, CA, 4752–4755. 10.1109/EMBC.2012.6347029 23366990

[B6] Bauer M. Klingauf U. (2006). Virtual-reality as a future training medium for civilian flight procedure training. *Paper Presented at the 2006 AIAA Modeling and Simulation Technologies Conference & Exhibit*, Keystone, CO.

[B7] Berger A. Pixa N. H. Steinberg F. Doppelmayr M. (2018). Brain oscillatory and hemodynamic activity in a bimanual coordination task following transcranial alternating current stimulation (tacs): a combined eeg-fnirs study. *Front. Behav. Neurosci.* 12:67. 10.3389/fnbeh.2018.00067 29720935 PMC5915568

[B8] Borwick C. Lal R. Lim L. W. Stagg C. J. Aquili L. (2020). Dopamine depletion effects on cognitive flexibility as modulated by tdcs of the dlpfc. *Brain Stimul.* 13 105–108. 10.1016/j.brs.2019.08.016 31494070 PMC7116421

[B9] Boto E. Holmes N. Leggett J. Roberts G. Shah V. Meyer S. S. (2018). Moving magnetoencephalography towards real-world applications with a wearable system. *Nature* 555 657–661. 10.1038/nature26147 29562238 PMC6063354

[B10] Brunoni A. R. Nitsche M. A. Bolognini N. Bikson M. Wagner T. Merabet L. (2012). Clinical research with transcranial direct current stimulation (tdcs): challenges and future directions. *Brain Stimul.* 5 175–195. 10.1016/j.brs.2011.03.002 22037126 PMC3270156

[B11] Cheng X. Li X. Hu Y. (2015). Synchronous brain activity during cooperative exchange depends on gender of partner: a fnirs-based hyperscanning study. *Hum. Brain Mapp.* 36 2039–2048. 10.1002/hbm.22754 25691124 PMC6869051

[B12] Choe J. Coffman B. A. Bergstedt D. T. Ziegler M. D. Phillips M. E. (2016). Transcranial direct current stimulation modulates neuronal activity and learning in pilot training. *Front. Hum. Neurosci.* 10:34. 10.3389/fnhum.2016.00034 PMC4746294 26903841

[B13] Coleman N. E. Pon S. (2013). Quality: performance improvement, teamwork, information technology and protocols. *Crit. Care Clin.* 29 129–151. 23537668 10.1016/j.ccc.2012.11.002

[B14] Colombo B. Anctil R. Balzarotti S. Biassoni F. Antonietti A. (2021). The role of the mirror system in influencing musicians’ evaluation of musical creativity: a tdcs study. *Front. Neurosci.* 15:624653. 10.3389/fnins.2021.624653 PMC8061779 33897346

[B15] Cui X. Bryant D. M. Reiss A. L. (2012). Nirs-based hyperscanning reveals increased interpersonal coherence in superior frontal cortex during cooperation. *Neuroimage* 59 2430–2437. 21933717 10.1016/j.neuroimage.2011.09.003 PMC3254802

[B16] Czeszumski A. Eustergerling S. Lang A. Menrath D. Gerstenberger M. Schuberth S. (2020). Hyperscanning: a valid method to study neural inter-brain underpinnings of social interaction. *Front. Hum. Neurosci.* 14:39. 10.3389/fnhum.2020.00039 32180710 PMC7059252

[B17] DaSilva A. F. Truong D. Q. DosSantos M. F. Toback R. L. Datta A. Bikson M. (2015). State-of-art neuroanatomical target analysis of high-definition and conventional tdcs montages used for migraine and pain control. *Front. Neuroanat.* 9:89. 10.3389/fnana.2015.00089 26236199 PMC4502355

[B18] Davis S. E. Smith G. A. (2019). Transcranial direct current stimulation use in warfighting: benefits, risks, and future prospects. *Front. Hum. Neurosci.* 13:114. 10.3389/fnhum.2019.00114 PMC6499187 31105538

[B19] Dodge L. E. Nippita S. Hacker M. R. Intondi E. M. Ozcelik G. Paul M. E. (2019). Impact of teamwork improvement training on communication and teamwork climate in ambulatory reproductive health care. *J. Healthc. Risk Manage*. 38 44–54. 10.1002/jhrm.21353 30212606

[B20] Doyle L. Kelliher F. Harrington D. (2020). Multi-level learning in public healthcare medical teams: the role of the social environment. *J. Health Organ. Manage.* 35 88–105. 10.1108/JHOM-05-2019-0135 33215478

[B21] Driskell T. Salas E. Driskell J. E. (2018). Teams in extreme environments: alterations in team development and teamwork. *Hum. Resour. Manage. Rev*. 28 434–449. 10.1016/j.hrmr.2017.01.002

[B22] Edelman B. J. Meng J. Suma D. Zurn C. Nagarajan E. Baxter B. S. (2019). Noninvasive neuroimaging enhances continuous neural tracking for robotic device control. *Sci. Robot.* 4:eaaw6844. 10.1126/scirobotics.aaw6844 31656937 PMC6814169

[B23] Enticott P. G. Arnold S. L. Fitzgibbon B. M. Hoy K. E. Susilo D. A. Fitzgerald P. B. (2012). Transcranial direct current stimulation (tdcs) of the inferior frontal gyrus disrupts interpersonal motor resonance. *Neuropsychologia* 50 1628–1631. 10.1016/j.neuropsychologia.2012.03.016 22465862

[B24] Fertonani A. Miniussi C. (2017). Transcranial electrical stimulation. *Neuroscientist* 23 109–123. 10.1177/1073858416631966 26873962 PMC5405830

[B25] Gozde C. (2017). *Design of a Wearable Fnirs Neuroimaging Device with an Internet-of-Things Architecture.* Master’s thesis. Kingston RI: University of Rhode Island.

[B26] Grover S. Nguyen J. A. Viswanathan V. Reinhart R. M. G. (2021). High-frequency neuromodulation improves obsessive–compulsive behavior. *Nat. Med.* 27 232–238. 10.1038/s41591-020-01173-w 33462447 PMC9331184

[B27] Guo H. Zhang Z. Da S. Sheng X. Zhang X. (2018). High-definition transcranial direct current stimulation (hd-tdcs) of left dorsolateral prefrontal cortex affects performance in balloon analogue risk task (bart). *Brain Behav*. 8:e00884. 10.1002/brb3.884 29484257 PMC5822580

[B28] Hamilton A. F. D. C. (2008). Emulation and mimicry for social interaction: a theoretical approach to imitation in autism. *Q. J. Exp. Psychol.* 61 101–115. 10.1080/17470210701508798 18038342

[B29] Hans J. E. Helsdingen A. S. Sluimer R. R. (2016). An empirical evaluation of transfer-of-training of two flight simulation games. *Simul. Gam.* 48 8–35. 10.1177/1046878116671057

[B30] Hari R. Kujala M. V. (2009). Brain basis of human social interaction: from concepts to brain imaging. *Physiol. Rev.* 89 453–479. 10.1152/physrev.00041.2007 19342612

[B31] He B. Sohrabpour A. Brown E. Liu Z. (2018). Electrophysiological source imaging: a noninvasive window to brain dynamics. *Annu. Rev. Biomed. Eng.* 20 171–196. 10.1146/annurev-bioeng-062117-120853 29494213 PMC7941524

[B32] Herrmann C. S. Rach S. Neuling T. Strüber D. (2013). Transcranial alternating current stimulation: a review of the underlying mechanisms and modulation of cognitive processes. *Front. Hum. Neurosci.* 7:279. 10.3389/fnhum.2013.00279 23785325 PMC3682121

[B33] Herrmann C. S. Strüber D. Helfrich R. F. Engel A. K. (2016). Eeg oscillations: from correlation to causality. *Int. J. Psychophysiol.* 103 12–21. 10.1016/j.ijpsycho.2015.02.003 25659527

[B34] Hogeveen J. Obhi S. S. Banissy M. J. Santiesteban I. Press C. Catmur C. (2015). Task-dependent and distinct roles of the temporoparietal junction and inferior frontal cortex in the control of imitation. *Soc. Cogn. Affect. Neurosci.* 10 1003–1009. 10.1093/scan/nsu148 25481003 PMC4483570

[B35] Hu Y. Wang Z. Song B. Pan Y. Cheng X. Zhu Y. (2021). How to calculate and validate inter-brain synchronization in a fnirs hyperscanning study. *J. Vis. Exp.* 175 1–16. 10.3791/62801 34570104

[B36] Hua X. Ke Long L. Ning H. (2018). Cooperation makes two less-creative individuals turn into a highly-creative pair. *Neuroimage* 172 527–537. 10.1016/j.neuroimage.2018.02.007 29427846

[B37] Jian L. W. C. (2017). Eview and prospect of cooperative combat of manned/unmanned aerial vehicle hybrid formation. *Aerospace Control* 35 90–96. 10.16804/j.cnki.issn1006-3242.2017.03.017

[B38] Katz B. Au J. Buschkuehl M. Abagis T. Zabel C. Jaeggi S. M. (2017). Individual differences and long-term consequences of tdcs-augmented cognitive training. *J. Cogn. Neurosci.* 29 1498–1508. 10.1162/jocn_a_01115 28253083

[B39] Ke Y. Wang N. Du J. Kong L. Liu S. Xu M. (2019). The effects of transcranial direct current stimulation (tDCS) on working memory training in healthy young adults. *Front. Hum. Neurosci.* 13:19. 10.3389/fnhum.2019.00019 30774590 PMC6367257

[B40] Khedr E. M. Salama R. H. Abdel Hameed M. Abo Elfetoh N. Seif P. (2019). Therapeutic role of transcranial direct current stimulation in Alzheimer disease patients: double-blind, placebo-controlled clinical trial. *Neurorehabil. Neural Res.* 33 384–394. 10.1177/1545968319840285 30940012

[B41] Kingsbury L. Hong W. (2020). A multi-brain framework for social interaction. *Trends Neurosci.* 43 651–666. 10.1016/j.tins.2020.06.008 32709376 PMC7484406

[B42] Kronberg G. Rahman A. Sharma M. Bikson M. Parra L. C. (2020). Direct current stimulation boosts hebbian plasticity *in vitro*. *Brain Stimul.* 13 287–301. 10.1016/j.brs.2019.10.014 31668982 PMC6989352

[B43] Lechler T. (2001). Social interaction: a determinant of entrepreneurial team venture success. *Small Business Economics*. 16 263–278. 10.1023/A:1011167519304

[B44] Li L. Wang H. Luo H. Zhang X. Zhang R. Li X. (2020). Interpersonal neural synchronization during cooperative behavior of basketball players: a fnirs-based hyperscanning study. *Front. Hum. Neurosci.* 14:169. 10.3389/fnhum.2020.00169 32733216 PMC7358650

[B45] Li R. Mayseless N. Balters S. Reiss A. L. (2021). Dynamic inter-brain synchrony in real-life inter-personal cooperation: a functional near-infrared spectroscopy hyperscanning study. *Neuroimage* 238:118263. 10.1016/j.neuroimage.2021.118263 34126210

[B46] Lipka R. Ahlers E. Reed T. L. Karstens M. I. Nguyen V. Bajbouj M. (2021). Resolving heterogeneity in transcranial electrical stimulation efficacy for attention deficit hyperactivity disorder. *Exp. Neurol.* 337:113586. 10.1016/j.expneurol.2020.113586 33382986

[B47] Liu D. Liu S. Liu X. Zhang C. Li A. Jin C. (2018). Interactive brain activity: review and progress on eeg-based hyperscanning in social interactions. *Front. Psychol.* 9:1862. 10.3389/fpsyg.2018.01862 30349495 PMC6186988

[B48] Liu T. Duan L. Dai R. Pelowski M. Zhu C. (2021). Team-work, team-brain: exploring synchrony and team interdependence in a nine-person drumming task via multiparticipant hyperscanning and inter-brain network topology with fnirs. *Neuroimage*. 237:118147. 10.1016/j.neuroimage.2021.118147 33984492

[B49] Lu H. Gong Y. Huang P. Zhang Y. You X. (2021). Effect of repeated anodal hd-tdcs on executive functions: evidence from a pilot and single-blinded fNIRS study. *Front. Hum. Neurosci.* 14:583730. 10.3389/fnhum.2020.583730 33536886 PMC7847848

[B50] Lu H. Liu Q. Guo Z. Zhou G. Zhang Y. Zhu X. (2020). Modulation of repeated anodal hd-tdcs on attention in healthy young adults. *Front. Psychol.* 11:564447. 10.3389/fpsyg.2020.564447 33329194 PMC7714753

[B51] Lu K. Hao N. (2019). When do we fall in neural synchrony with others? *Soc. Cogn. Affect. Neurosci*. 14 253–261. 10.1093/scan/nsz012 30753646 PMC6413689

[B52] Lu K. Xue H. Nozawa T. Hao N. (2019). Cooperation makes a group be more creative. *Cereb. Cortex* 29 3457–3470. 10.1093/cercor/bhy215 30192902

[B53] Mainieri A. G. Heim S. Straube B. Binkofski F. Kircher T. (2013). Differential role of the mentalizing and the mirror neuron system in the imitation of communicative gestures. *Neuroimage* 81 294–305. 10.1016/j.neuroimage.2013.05.021 23684882

[B54] Martin S. R. Emich K. J. Mcclean E. J. Woodruff C. T. (2022). Keeping teams together: how ethical leadership moderates the effects of performance on team efficacy and social integration. *J. Bus. Ethics* 176 127–139. 10.1007/s10551-020-04685-0

[B55] Mayseless N. Hawthorne G. Reiss A. L. (2019). Real-life creative problem solving in teams: fnirs based hyperscanning study. *Neuroimage* 203:116161. 10.1016/j.neuroimage.2019.116161 31493532

[B56] Meiran N. Pereg M. Givon E. Danieli G. Shahar N. (2016). The role of working memory in rapid instructed task learning and Intention-Based reflexivity: an individual differences examination. *Neuropsychologia* 90 180–189. 10.1016/j.neuropsychologia.2016.06.037 27374319

[B57] Meng Yuan W. Ping L. Juan Z. Yu Tao X. Niu H. J. Zhen Y. (2018). Concurrent mapping of brain activation from multiple subjects during social interaction by hyperscanning: a mini-review. *Quant. Imaging Med. Surgery* 8 819–837. 10.21037/qims.2018.09.07 30306062 PMC6177358

[B58] Metuki N. Sela T. Lavidor M. (2012). Enhancing cognitive control components of insight problems solving by anodal tdcs of the left dorsolateral prefrontal cortex. *Brain Stimul.* 5 110–115. 10.1016/j.brs.2012.03.002 22483547

[B59] Min C. (2013). Survey on modeling of target allocation for formation cooperative combat. *Electron. Opt. Control* 20 53–58.

[B60] Moussavi Z. Kimura K. Kehler L. de Oliveira Francisco C. Lithgow B. (2021). A novel program to improve cognitive function in individuals with dementia using transcranial alternating current stimulation (tacs) and tutored cognitive exercises. *Front. Aging* 2:3. 10.3389/fragi.2021.632545 PMC9261296 35822057

[B61] Nguyen T. Hoehl S. Vrtièka P. (2021a). A guide to parent-child fnirs hyperscanning data processing and analysis. *Sens. Basel* 21:4075. 10.3390/s21124075 34199222 PMC8231828

[B62] Nguyen T. Schleihauf H. Kungl M. Kayhan E. Hoehl S. Vrtièka P. (2021b). Interpersonal neural synchrony during father–child problem solving: an fnirs hyperscanning study. *Child Dev.* 92 e565–e580. 10.1111/cdev.13510 33426676 PMC8451924

[B63] Nikolin S. Huggins C. Martin D. Alonzo A. Loo C. (2019). Adverse events associated with repeated sessions of tdcs: a systematic review and meta-analysis. *Brain Stimul.* 12 483. 10.1016/j.brs.2018.12.577 29169814

[B64] Nissim N. R. O Shea A. Indahlastari A. Telles R. Richards L. Porges E. (2019). Effects of in-Scanner bilateral frontal tDCS on functional connectivity of the working memory network in older adults. *Front. Aging Neurosci.* 11:51. 10.3389/fnagi.2019.00051 30930766 PMC6428720

[B65] Nitsche M. A. Liebetanz D. Tergau F. Paulus W. (2002). Modulation of cortical excitability by transcranial direct current stimulation. *Nervenarzt* 73 332–335. 10.1007/s00115-002-1272-9 12040980

[B66] Novembre G. Iannetti G. D. (2021a). Hyperscanning alone cannot prove causality. Multibrain stimulation can. *Trends Cogn. Sci.* 25 96–99. 10.1016/j.tics.2020.11.003 33293210 PMC7994246

[B67] Novembre G. Iannetti G. D. (2021b). Proving causality in hyperscanning: multibrain stimulation and other approaches: response to moreau and dumas. *Trends Cogn. Sci.* 25 544–545. 10.1016/j.tics.2021.03.013 33941464

[B68] Novembre G. Knoblich G. Dunne L. Keller P. E. (2017). Interpersonal synchrony enhanced through 20 hz phase-coupled dual brain stimulation. *Soc. Cogn. Affect. Neurosci.* 12 662–670. 10.1093/scan/nsw172 28119510 PMC5390732

[B69] Oberman L. M. Pineda J. A. Ramachandran V. S. (2007). The human mirror neuron system: a link between action observation and social skills. *Soc. Cogn. Affect. Neurosci.* 2 62–66. 10.1093/scan/nsl022 18985120 PMC2555434

[B70] Ohlander U. Alfredson J. Riveiro M. Falkman G. (2016a). “A teamwork model for fighter pilots,” in *Proceedings of the 2016 International Conference on Engineering Psychology and Cognitive Ergonomics*, Toronto, ON.

[B71] Ohlander U. Alfredson J. Riveiro M. Falkman G. (2016b). “Elements of team effectiveness: a qualitative study with pilots,” in *Proceedings of the 2016 IEEE International Multi-disciplinary Conference on Cognitive Methods in Situation Awareness & Decision Support*, San Diego, CA.

[B72] Ohlander U. Alfredson J. Riveiro M. Falkman G. (2018). *Informing the Design of Fighter Aircraft Cockpits Using a Teamwork Perspective.* Cham: Springer.

[B73] Ohlander U. Alfredson J. Riveiro M. Falkman G. (2019). Fighter pilots’ teamwork: a descriptive study. *Ergonomics* 62 880–890. 31002026 10.1080/00140139.2019.1596319

[B74] Ohlander U. Alfredson J. Riveiro M. Xf G. Falkman R. (2009). *The Use of Structural Equation Modeling to Describe the Effect of Operator Functional State on Air-to-Air Engagement Outcomes.* Linköping: Linköping University Electronic Press.

[B75] Pa Rlikar R. Sreeraj V. S. Shivakumar V. Narayanaswamy J. C. Venkatasubramanian G. (2021). High definition transcranial direct current stimulation (hd-tdcs): a systematic review on the treatment of neuropsychiatric disorders. *Asian J. Psychiatry* 56:102542. 10.1016/j.ajp.2020.102542 33486461

[B76] Pan Y. Cheng X. Zhang Z. Li X. Hu Y. (2017). Cooperation in lovers: an fnirs-based hyperscanning study. *Hum. Brain Mapp.* 38 831–841. 10.1002/hbm.23421 27699945 PMC6867051

[B77] Pan Y. Novembre G. Song B. Zhu Y. Hu Y. (2021). Dual brain stimulation enhances interpersonal learning through spontaneous movement synchrony. *Soc. Cogn. Affect. Neurosci.* 16 210–221. 10.1093/scan/nsaa080 32591830 PMC7812617

[B78] Peled-Avron L. Glasner L. Gvirts H. Z. Shamay-Tsoory S. G. (2019). The role of the inferior frontal gyrus in vicarious social touch: a transcranial direct current stimulation (tdcs) study. *Dev. Cogn. Neurosci.* 35 115–121. 10.1016/j.dcn.2018.04.010 29773509 PMC6968961

[B79] Pennisi E. (2017). Why wolves are better team players than dogs. *Science.* 10.1126/science.aar2313

[B80] Prete G. D’Anselmo A. Tommasi L. Brancucci A. (2017). Modulation of illusory auditory perception by transcranial electrical stimulation. *Front. Neurosci.* 11:351. 10.3389/fnins.2017.00351 28676740 PMC5476865

[B81] Prete G. D’Anselmo A. Tommasi L. Brancucci A. (2018). Modulation of the dichotic right ear advantage during bilateral but not unilateral transcranial random noise stimulation. *Brain Cogn*. 123 81–88. 10.1016/j.bandc.2018.03.003 29547746

[B82] Redcay E. Dodell-Feder D. Pearrow M. J. Mavros P. L. Kleiner M. Gabrieli J. D. E. (2010). Live face-to-face interaction during fmri: a new tool for social cognitive neuroscience. *Neuroimage* 50 1639–1647. 10.1016/j.neuroimage.2010.01.052 20096792 PMC2849986

[B83] Reed T. Cohen Kadosh R. (2018). Transcranial electrical stimulation (tes) mechanisms and its effects on cortical excitability and connectivity. *J. Inherit. Metab. Dis.* 41 1123–1130. 10.1007/s10545-018-0181-4 30006770 PMC6326965

[B84] Reindl V. Konrad K. Gerloff C. Kruppa J. A. Bell L. Scharke W. (2019). Conducting hyperscanning experiments with functional near-infrared spectroscopy. *J. Vis. Exp.* 143:e58807. 10.3791/58807 30735168

[B85] Reinhart R. M. G. Nguyen J. A. (2019). Working memory revived in older adults by synchronizing rhythmic brain circuits. *Nat. Neurosci.* 22 820–827. 10.1038/s41593-019-0371-x 30962628 PMC6486414

[B86] Riddle J. McFerren A. Frohlich F. (2021). Causal role of cross-frequency coupling in distinct components of cognitive control. *Prog. Neurobiol.* 202:102033. 10.1016/j.pneurobio.2021.102033 33741402 PMC8184612

[B87] Salas E. Dickinson T. L. Converse S. A. Tannenbaum S. I. (1992). “Toward an understanding of team performance and training,” in *Teams: Their Training and Performance*, eds Swezey R. W. Salas E. (Norwood, NJ: Ablex Publishing), 3–29.

[B88] Salas E. Sims D. E. Burke C. S. (2005). Is there a “big five” in teamwork? *Small Group Res*. 36 555–599. 10.1177/1046496405277134

[B89] Scheel D. Packer C. (1991). Group hunting behaviour of lions: a search for cooperation. *Anim. Behav.* 41 697–709. 10.1016/S0003-3472(05)80907-8

[B90] Seeber M. Cantonas L. Hoevels M. Sesia T. Visser-Vandewalle V. Michel C. M. (2019). Subcortical electrophysiological activity is detectable with high-density EEG source imaging. *Nat. Commun.* 10:753. 10.1038/s41467-019-08725-w 30765707 PMC6376013

[B91] Seok D. Lee S. Kim M. Cho J. Kim C. (2021). Motion artifact removal techniques for wearable EEG and PPG sensor systems. *Front. Electron.* 2:85513. 10.3389/felec.2021.685513

[B92] Shibata H. Inui T. Ogawa K. (2011). Understanding interpersonal action coordination: an fmri study. *Exp. Brain Res.* 211 569–579. 10.1007/s00221-011-2648-5 21509492

[B93] Shin Y. I. Foerster Á Nitsche M. A. (2015). Transcranial direct current stimulation (tdcs) – application in neuropsychology. *Neuropsychologia* 69 154–175. 10.1016/j.neuropsychologia.2015.02.002 25656568

[B94] Stowers K. Brady L. L. MacLellan C. Wohleber R. Salas E. (2021). Improving teamwork competencies in human-machine teams: perspectives from team science. *Front. Psychol.* 12:590290. 10.3389/fpsyg.2021.590290 34108903 PMC8181721

[B95] Sun B. Xiao W. Lin S. Shao Y. Li W. Zhang W. (2021). Cooperation with partners of differing social experience: an fnirs-based hyperscanning study. *Brain Cogn*. 154:105803. 10.1016/j.bandc.2021.105803 34689103

[B96] Svensson Å. Ohlander U. Lundberg J. (2020). Design implications for teamwork in atc. *Cogn. Technol. Work* 22 409–426. 10.1007/s10111-019-00579-y

[B97] Szymanski C. Müller V. Brick T. R. von Oertzen T. Lindenberger U. (2017). Hyper-transcranial alternating current stimulation: experimental manipulation of inter-brain synchrony. *Front. Hum. Neurosci.* 11:539. 10.3389/fnhum.2017.00539 29167638 PMC5682643

[B98] Tabassi A. A. Ramli M. Bakar A. H. A. (2012). Effects of training and motivation practices on teamwork improvement and task efficiency: the case of construction firms. *Int. J. Proj. Manage*. 30 213–224. 10.1016/j.ijproman.2011.05.009

[B99] Takeuchi N. Izumi S. (2021). Motor learning based on oscillatory brain activity using transcranial alternating current stimulation: a review. *Brain Sci*. 11:1095. 10.3390/brainsci11081095 34439714 PMC8392205

[B100] Teresa W. Marisa B. (2015). Fnirs in the developmental sciences. *Wiley Interdiscip. Rev. Cogn. Sci*. 6 263–283. 10.1002/wcs.1343 26263229 PMC4979552

[B101] Tomer Y. Ng Gong M. Keller M. J. Southern W. Kitsis E. A. Kajita G. R. (2021). Teamwork and leadership under fire at the epicenter of the covid-19 epidemic in the bronx. *Front. Med.* 8:610100. 10.3389/fmed.2021.610100 33816518 PMC8012527

[B102] Toppi J. Borghini G. Petti M. He E. J. De Giusti V. He B. (2016). Investigating cooperative behavior in ecological settings: an eeg hyperscanning study. *PLoS One* 11:e154236. 10.1371/journal.pone.0154236 27124558 PMC4849782

[B103] Turski C. A. Kessler-Jones A. Chow C. Hermann B. Hsu D. Jones J. (2017). Extended multiple-field high-definition transcranial direct current stimulation (hd-tdcs) is well tolerated and safe in healthy adults. *Restor. Neurol. Neurosci*. 35 631–642. 10.3233/RNN-170757 29172010 PMC5730273

[B104] Villafaina S. Fuentes-García D. Gusi N. Tornero-Aguilera J. F. Clemente-Suárez V. J. (2021). Psychophysiological response of military pilots in different combat flight maneuvers in a flight simulator. *Physiol. Behav.* 238:113483. 10.1016/j.physbeh.2021.113483 34097973

[B105] Xu C. Bryant D. M. Reiss A. L. (2012). Nirs-based hyperscanning reveals increased interpersonal coherence in superior frontal cortex during cooperation. *Neuroimage* 59 2430–2437. 21933717 10.1016/j.neuroimage.2011.09.003 PMC3254802

[B106] Zeng L. Guo M. Wu R. Luo Y. Wei P. (2022). The effects of electroencephalogram Feature-Based transcranial alternating current stimulation on working memory and electrophysiology. *Front. Aging Neurosci.* 14:828377. 10.3389/fnagi.2022.828377 35360204 PMC8961031

[B107] Zhao H. Cheng T. Zhai Y. Long Y. Wang Z. Lu C. (2021). How mother–child interactions are associated with a child’s compliance. *Cereb. Cortex* 31 4398–4410. 10.1093/cercor/bhab094 33895811

[B108] Zhiqiang H. (2015). *Research on the Incentive of Air Force Flying Cadets Who’Sundergraduate Education for Academic Credential.* Changchun: Northeast Normal University.

